# Spatial Association Pattern of Air Pollution and Influencing Factors in the Beijing–Tianjin–Hebei Air Pollution Transmission Channel: A Case Study in Henan Province

**DOI:** 10.3390/ijerph17051598

**Published:** 2020-03-02

**Authors:** Jianhui Qin, Suxian Wang, Linghui Guo, Jun Xu

**Affiliations:** 1 School of Business and Administration, Henan Polytechnic University, Jiaozuo 454000, Henan, China; qinjianhui@hpu.edu.cn; 2 Emergency Management School, Henan Polytechnic University, Jiaozuo 454000, Henan, China; 2277410@163.com; 3 School of Surveying and Land Information Engineering, Henan Polytechnic University, Jiaozuo 454000, Henan, China; 4 School of Business, Jiangsu Normal University, Xuzhou 221116, Jiangsu, China; lmmxjxj@163.com

**Keywords:** air pollution, changing characteristic, spatial association pattern, influencing factors, Henan Province

## Abstract

The Beijing–Tianjin–Hebei (BTH) air pollution transmission channel and its surrounding areas are of importance to air pollution control in China. Based on daily data of air quality index (AQI) and air pollutants (PM_2.5_, PM_10_, SO_2_, NO_2_, CO, and O_3_) from 2015 to 2016, this study analyzed the spatial and temporal characteristics of air pollution and influencing factors in Henan Province, a key region of the BTH air pollution transmission channel. The result showed that non-attainment days and NAQI were slightly improved at the provincial scale during the study period, whereas that in Hebi, Puyang, and Anyang became worse. PM_2.5_ was the largest contributor to the air pollution in all cities based on the number of non-attainment days, but its mean frequency decreased by 21.62%, with the mean occurrence of O_3_ doubled. The spatial distribution of NAQI presented a spatial agglomeration pattern, with high-high agglomeration area varying from Jiaozuo, Xinxiang, and Zhengzhou to Anyang and Hebi. In addition, the NAQI was negatively correlated with sunshine duration, temperature, relative humidity, wind speed, and positively to atmospheric pressure and relative humidity in all four clusters, whereas relationships between socioeconomic factors and NAQI differed among them. These findings highlight the need to establish and adjust regional joint prevention and control of air pollution as well as suggest that it is crucially important for implementing effective strategies for O_3_ pollution control.

## 1. Introduction

With rapid economic growth and dramatic increase in energy consumption, China has been experiencing serious environment problems related to air pollution, leading to adverse impacts on human health [1,2] and socioeconomic development [3]. The air pollution issue has attracted increasing attention of all aspects of the society [4,5]. In recent years, a number of studies have investigated the spatial and temporal distribution of air pollution and pollutants in China [6,7,8,9]. For instance, Xu et al. and Wang et al. addressed the variations of air quality and air pollutants in 31 provincial capital cities [6,8]. Yan et al. analyzed the distribution of PM_2.5_ concentrations in the Beijing–Tianjin–Hebei (BTH) metropolis circle and highlighted its increase from late autumn to early winter with an obvious spatial range from southeast to northwest [10]. However, most studies have mainly focused on the megacity clusters such as the BTH metropolis circle [10,11], Pearl River Delta (PRD) [12], Yangtze River Delta (YRD) [13,14], and the capital cities in China [15]. Studies have indicated that, due to vast territories, complex air pollution emissions, and environmental conditions, regional differences are remarkable for ambient air quality [16], composition of air pollutants [17], and spatiotemporal processes [14]. Therefore, it is difficult to extrapolate existing findings to other regions, and advancing our understanding of variations in air pollution in different regions is thus necessary and helpful for supporting the establishment of region-oriented air quality control measures. Moreover, recent studies have suggested that the degree of air pollution in a city is closely correlated with that of surrounding cities, with the feature of “be bound together for good or ill” [9,18,19]. Hence, clarifying the spatial association pattern and spatial spillover effects of air pollution among cities is of importance in improving our strategies of regional air pollution prevention and control. 

Numerous studies have shown that meteorological conditions a play key role in the formation and variation of air pollution [13,16]. An increase in temperature could diffuse air pollution and dilute pollutant concentrations [20], and a high atmospheric pressure usually restricts the dispersion and dilution of pollutants [16]. Air quality may be better on days with more sunshine, higher wind speed, temperature, relative humidity, and precipitation [8]. However, recent studies have suggested that the influence of meteorological conditions on air pollution is much more complex and differs with time and region scales [21,22]. For instance, air pollution was strongly negatively correlated with temperature in southern China, while many cities in northeastern and northern China showed a positive correlation [22]. On the other hand, it has been well documented that socioeconomic factors such as per capita GDP, population density, urbanization level, proportion of secondary industry, car ownership, and energy structure are important internal drivers of air pollution, but their influence direction and intensity also vary geographically due to the regional development and resource environment status [23,24]. Thus, it is necessary to quantify the spatial heterogeneity influences of meteorological and socioeconomic factors on air pollution. 

Henan Province, a crucial region of the BTH air pollution transmission channel, has been one of the most heavily polluted areas in China [12], and its provincial capital city, Zhengzhou, was listed among “the top ten worst cities for air quality” in China during 2013–2017 [25]. However, our understanding of the air pollution in this region is fairly limited, which further restricts our ability to implement effective air pollution prevention and control policies. Therefore, in this study, we analyzed the spatiotemporal variation of urban air quality index for days that exceeded the ambient air quality standards and major pollutants in Henan Province using newly released monitoring data. In addition, we quantified the spatial patterns of air pollution and influencing factors to enhance our understanding of the underlying mechanisms, which can be used as a reference for establishing region-oriented air quality control measures.

## 2. Materials and Methods

### 2.1. Study Area 

**Figure 1:**
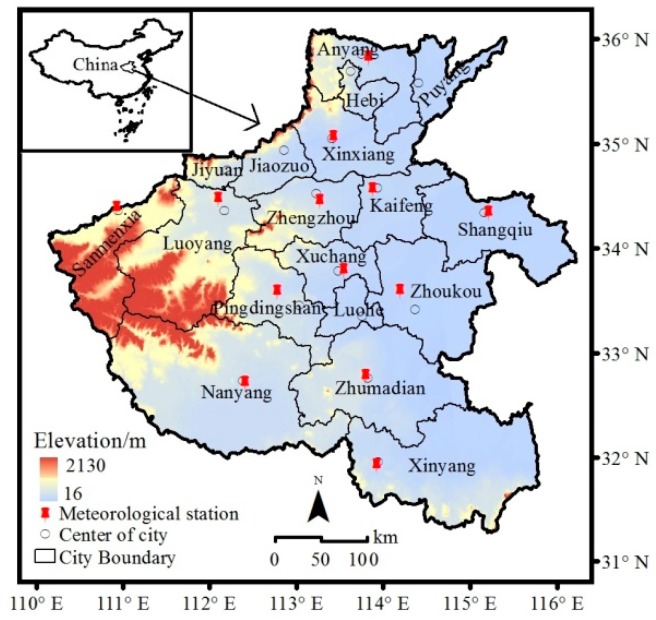
Map of Henan Province in China including the locations of the 18 cities and 13 meteorological stations.

Consisting of 18 cities and covering an area of approximately 167,000 km^2^, Henan Province is located in the key region of the Beijing–Tianjin–Hebei air pollution transmission channel (Figure 1). The elevation of the province increases from east to west (from 16 m to 2130 m, respectively). This region experiences a continental monsoon climate with annual mean rainfall of 408–1296 mm and annual mean temperature of 10.5–16.7 °C. Henan Province is the most populated province, with more than 120 million people, and is one of the most polluted areas in China [26]. 

### 2.2. Data Sources

Daily datasets of the air quality index (AQI) and six major pollutants (PM_2.5_, PM_10_, CO, SO_2_, NO_2_, and O_3_) for the 18 cities in Henan Province from March 1, 2015 to February 28, 2017 were collected from the website of the Henan Bureau of Environmental Protection [27]. According to the newly air quality standard passed by the Ministry of Environmental Protection and the State General Administration of Quality Supervision Inspection and Quarantine of China, Henan Province has gradually started publishing air quality data of the six criteria pollutants for its 18 cities through its website since 2014. This latest database is essential for providing detailed information about the air pollution situation and can help citizens understand how local air quality has changed over time. In the data period of this study, there were three days with missing records in 2015, and two days with missing records in 2016. Thus, the corresponding records on these five days in each year were removed. As an integrated index, the AQI is defined as the maximum of the indices for the six criterion pollutants [26]. The pollutant with the highest index value is referred to as the “major pollutant”, if the AQI is above 50. Daily air quality is classified into six grades based on the AQI value: excellent (0–50), good (51–100), mild pollution (101–150), moderate pollution (151–200), heavy pollution (201–300), and serious pollution (>300). In this study, only the period of non-attainment days (defined as days with an AQI higher than 100) was used for analysis, and the AQI for these non-attainment days was denoted as the NAQI.

Daily meteorological data were obtained from the China Meteorological Data Sharing Service System [28]. These data included daily maximum instantaneous wind speed (MWS, m/s) and direction (MWD), average temperature (AT, °C), average atmospheric pressure (AAP, hPa), and sunshine hours (SH, h), average relative humidity (ARH, %), precipitation (P, mm), and average (AST, °C), and maximum surface temperature (MST, °C). The 13 meteorological stations are shown in Figure 1. For cities without an available meteorological station, the city’s meteorological dataset was substituted with a dataset from an adjacent station. For instance, the meteorological data of Puyang and Hebi were replaced by those of Anyang, those of Jiaouzo were replaced by those of Xinxiang, those of Jiyuan were replaced by those of Luoyang, and those of Luohe were replaced by those of Xuchang. The socioeconomic datasets, which include civil vehicle possession, energy consumption, the floor space of buildings, and economic development for each city were retrieved from the statistical yearbooks of Henan Province for 2016 and 2017 [29]. To eliminate any possible effects of differences in regional area, we used per unit area or per capita socioeconomic data instead of the total amount. To further determine the influences of specific socioeconomic activities, we included four additional types of socioeconomic indicators: per unit area possession of a civil vehicle (PCV, vehicles/km^2^), passenger vehicle (PPV, vehicles/km^2^), large passenger vehicle (PLPV, vehicles/km^2^), sedan (PS, vehicles/km^2^), truck (PT, vehicles/km^2^), heavy truck (PHT, vehicles/km^2^), and ordinary truck (POT, vehicles/km^2^); per unit area total energy consumption (PTEC, tons of standard coal/km^2^), consumption of coal (PCL, tons of standard coal/km^2^), coke (PCK, tons of standard coal/km^2^), diesel fuel (PDF, tons of standard coal/km^2^), heat (PH, kJ/km^2^), and electricity (PE, kWh/km^2^) of industrial enterprises above a designated size; per unit area floor space under construction (PFSCI, m^2^/km^2^) and floor space under completion (PFSCII, m^2^/km^2^); and the proportion of primary industry (PPI, %), proportion of secondary industry (PSI, %), proportion of tertiary industry (PTI, %), per capita gross domestic product (PGDP, yuan per capita), value-added by industry (PVAI, yuan per capita), and value-added by construction (PVAC, yuan per capita). These indicators were selected for following reasons: first, their importance has been addressed by the previous studies [14,19,24,30]; second, these indicators can comprehensively reflect the regional socioeconomic situation [31].

### 2.3. Spatial Association Pattern

Moran’s I is a widely used indicator of spatial association pattern [32]. To measure the spatial autocorrelation of the NAQI, the global Moran’s I ranging from −1 to 1, was calculated using the average NAQI in 2015 and 2016. When global Moran’s I is significantly bigger than 0, it shows a positive spatial correlation; when global Moran’s I is significantly lower than 0, it denotes a negative spatial correlation; “0” implies perfect spatial randomness [18]. Meanwhile, the local Moran’s I of the average NAQI in 2015 and 2016 was used for identifying local spatial cluster patterns and spatial outliers. A high positive local Moran’s I implies that the location is a spatial cluster, high–high cluster (H–H) or low–low cluster (L–L), while a high negative local Moran’s I indicates a potential spatial outlier, high–low (H–L) or low–high (L–H) outlier. Moran’s I analysis was performed using ArcGIS version 10.2. Given the importance of non-attainment days for air pollution exposure, a hierarchical clustering analysis was conducted by combining the mean seasonal NAQI and seasonal non-attainment days in each year of 2015 and 2016 for the 18 cities of Henan Province to investigate their spatial association pattern. The hierarchical clustering was performed with SPSS software version 16.0 using the Euclidean distance of the Z-score standardized variables to measure dissimilarities and Ward’s criterion to construct the hierarchical tree [33]. 

### 2.4. Pearson Correlation Analysis

To examine factors influencing the spatiotemporal variations of the NAQI, we examined the socioeconomic factors at the city level and the daily meteorological variables within the city level. Relationships were estimated according to the cluster analysis result to provide insights pertaining to the spatial heterogeneity of the influence factors. We performed this analysis using Pearson correlation coefficients calculated with SPSS version 16.0 software. 

## 3. Results and Discussion

### 3.1. Characteristics of Air Quality

#### 3.1.1. Variations of NAQI

**Figure 2:**
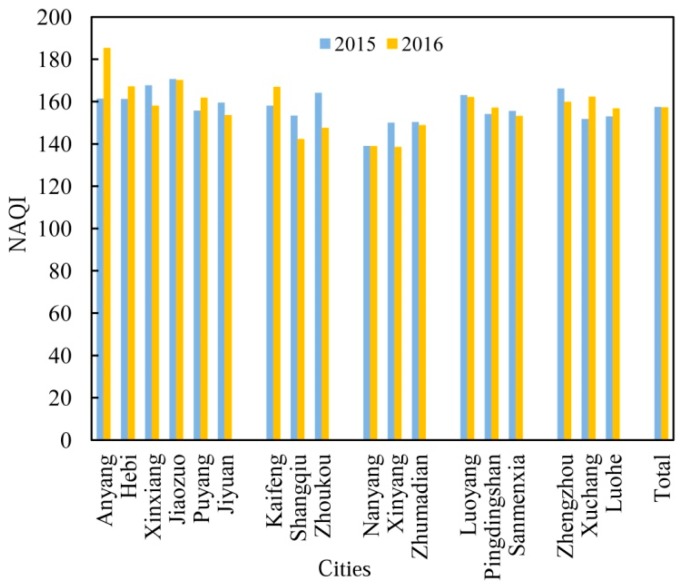
Variations in annual average air quality index (AQI) on non-attainment days at the city and province scales in 2015–2016.

The NAQI in Henan Province exhibited substantial spatiotemporal variations (Figure 2). Generally, cities with low NAQI values were located in the south (e.g., in Nanyang, Xinyang, and Zhumadian), whereas northern and central regions had relatively high NAQI values, which is consistent with the results of previous studies [2,25]. This gradient may have been caused by two possible reasons. The first reason might be due to the fact that the northern part of Henan Province is more economically dependent on mineral resources and industry than the southern part. For instance, the energy consumption per unit GDP in some resource-based cities in northern Henan such as Anyang, Hebi, and Jiaozuo is higher than the provincial and national average, which may result in high emissions of air pollutants [34]. Second, this can be related to changes in the meteorological conditions from south to north. The cities with lower temperatures in the north have been suggested to have worse air quality due to the vertical and horizontal turbulence [8]. Compared with 2015, the annual average NAQI in 2016 showed a slight decline at the province scale. However, the variations of NAQI in Anyang, Xuchang, Kaifeng, Puyang, and Hebi demonstrated obvious increases of 14.85%, 6.97%, 5.61%, 3.92%, and 3.69%, respectively, whereas the air quality of the three southern cities with the lowest NAQI values improved. This highlights the need to improve air quality through new effective efforts in high pollution cities. In addition, monthly NAQI was highest in winter (December–February) and lowest in summer (June–August), with an obvious decrease from May to October and in December, and a slight increase in other months from 2015 to 2016. This seasonal variation in the NAQI may be mainly related to the typical monsoon climate in Henan Province. For example, in winter and spring, the atmosphere is steady in structure with a low temperature, which is not conducive to diffuse air pollutants [31]. Meanwhile, the amount of pollutant emissions greatly increases due to coal combustion for domestic heating and dust weather at this time [8]. The combination of emissions and unfavorable weather conditions inevitably results in serious air pollution.

#### 3.1.2. Features of Non-Attainment Days

**Figure 3:**
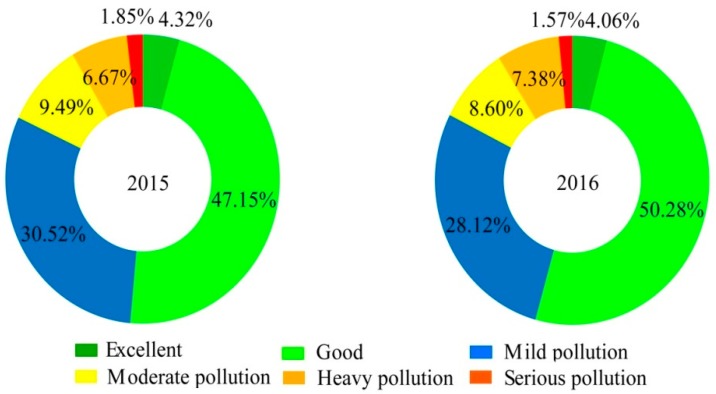
Proportion of days with different air quality levels at the province scale in 2015–2016.

The pie charts in Figure 3 show the proportions of days having a specific grade of daily air quality in Henan Province for the two years. On average, there were 174.72 non-attainment days in 2015, representing an air quality non-attainment rate of 48.53%. In particular, the number of days with mild and moderate pollution formed the main portion (144.05 days) and the days with heavy and serious pollution (30.67 days) accounted for about 8.51% of the total number of days of the year. Comparably, the number of non-attainment days showed a decline by 10.33 days in 2016, which had a non-attainment rate of 45.66%. Although the days with mild and moderate pollution still constituted the majority of the air pollution days, the number of these days reduced greatly to 132.17, whereas the number of heavy pollution and serious pollution days increased slightly to 32.22. 

**Figure 4:**
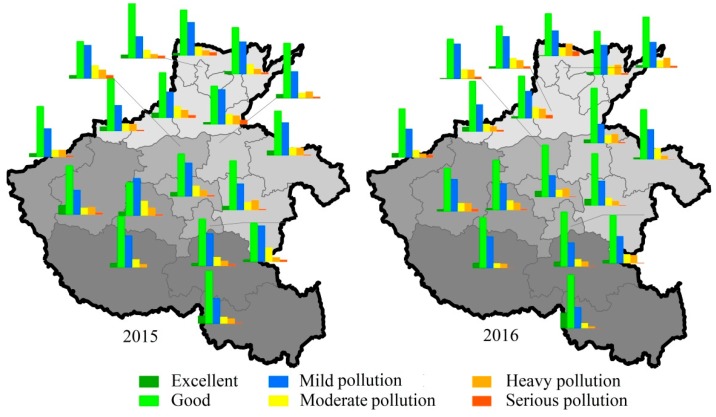
Proportions of days with different air quality levels at the city scale in 2015–2016. The grey color changes from light to deep denote north, east, center, west, and south Henan Province, respectively.

Substantial differences in air quality non-attainment days were observed among cities (Figure 4). Compared with the case in 2015, 12 of the 18 cities showed a decrease in non-attainment days, with the highest reductions in Pingdingshan (58 days), Xuchang (47 days), Luohe (41 days), and Zhumadian (39 days). In contrast, Luoyang (42 days), Hebi (30 days), Jiaozuo (17 days), Puyang (eight days), Anyang (seven days), and Jiyuan (three days) exhibited an increase in non-attainment days. Further analysis revealed that the increase in non-attainment days over the six cities, except Luoyang and Jiyuan, was mainly due to the increase in heavy pollution and serious pollution days, while the decline of non-attainment days in the other cities resulted from changes in mild and moderate pollution days.

#### 3.1.3. Changes in Major Pollutants

**Figure 5:**
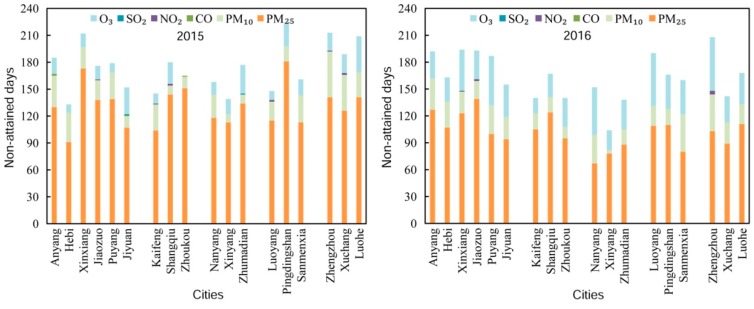
Contributions of the six pollutants as the major pollutant on non-attainment days at the city scale in 2015–2016.

Figure 5 illustrates the major pollutants on the non-attainment days at the city scale during the study period. As is shown, PM_2.5_ was the major pollutant on 66% or more of non-attainment days in all cities in 2015, with the highest rate in Zhoukou (91.52%) and lowest rate in Zhengzhou (66.20%). In contrast, although PM_2.5_ was the most frequent major pollutant in all cities in 2016, its mean frequency decreased from 75.01% to 62.49% of non-attainment days, with the highest rate of 74.25% occurred only in Shangqiu. Meanwhile, O_3_, as the second most frequent major pollutant extended from six cities (Jiyuan, Shangqiu, Xinyang, Zhumadian, Pingdingshan, and Luohe) to 14 of the 18 cities (except Anyang, Hebi, Kaifeng, and Sanmenxia) during 2015–2016. The mean occurrence of O_3_ as the major pollutant in Henan Province doubled, while the mean frequency of PM_10_ as the major pollutant showed a minor increase from 14.02% to 14.70% of non-attainment days. Additionally, occurrences of CO, NO_2_, and SO_2_ as the major pollutant were much less frequent. 

Previous study has suggested that PM_2.5_ was the most frequent major pollutant in north regions of China, accounting for about 66.5% of non-attainment days in 2013 [6], and addressed that the PM_2.5_ concentration in this region decreased sharply [35,36], possibly even became faster from 2015 to 2017 [37]. These findings are quite correspondent to the results from our work, which imply that control measures for power plants, floating dusts, and industries in Henan Province have become effective. The increased surface O_3_ pollution over the same time can be partly ascribed to reduced PM_2.5_ pollution, because PM_2.5_ scavenges hydroperoxy and NOx radicals, and thus speed up O_3_ generation [38]. In addition, it can be caused by volatile organic compounds and NOx emissions derived from fuel combustion and industrial sources [39], and biogenic sources in the context of global warming. The shift in major air pollutants indicates a new challenge for urban air quality improvement, and that additional efforts are required to control the formation of secondary pollutants.

### 3.2. Spatial Association Pattern

**Figure 6:**
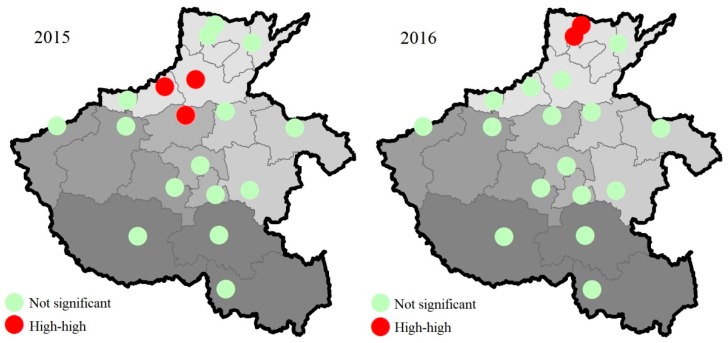
Local spatial autocorrelation model of the mean NAQI in 2015–2016. The grey color changes from light to deep denote north, east, center, west and south Henan Province, respectively.

The results of the global Moran’s I and local Moran’s I analysis are illustrated in Figure 6. Significant positive spatial autocorrelation was observed for mean NAQI in 2015 and 2016, with global Moran’s I values of 0.42 (*p* = 0.003), and 0.33 (*p* = 0.016), respectively, indicating that air quality is strongly affected by adjacent cities. From the local Moran’s I, a large high–high spatial cluster was found in Jiaozuo, Xinxiang, and Zhengzhou in 2015, and in Anyang and Hebi in 2016 (Figure 6). Similarly, based on the AQI for 338 cities in 2016, Sun et al. demonstrated that air quality had a remarkably significant spatial agglomeration effect in China, with the high–high agglomeration area mainly located in the north China Plain [9]. This is mainly caused by natural air flows on the flat terrain, industrial transfers, and product trades between neighboring cities [40]. This underscores the need for establishing and adjusting regional mechanisms for the joint prevention and control of air pollution.

**Figure 7:**
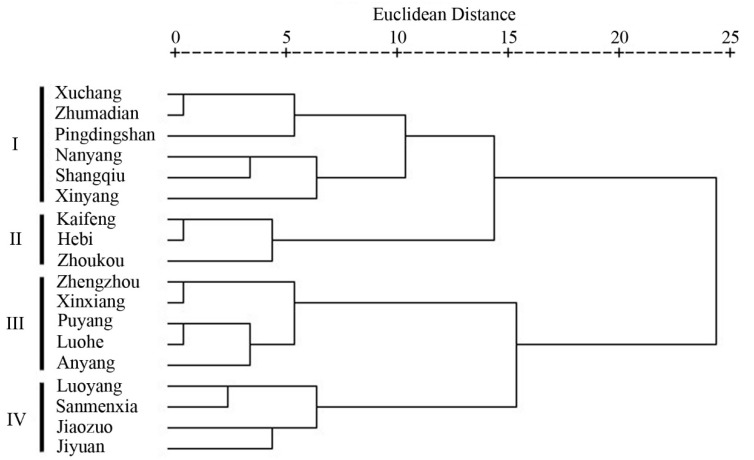
Dendrogram based on the Z-scored air quality index on non-attainment days (NAQI) and non-attainment days of 18 cities in 2015–2016.

**Figure 8:**
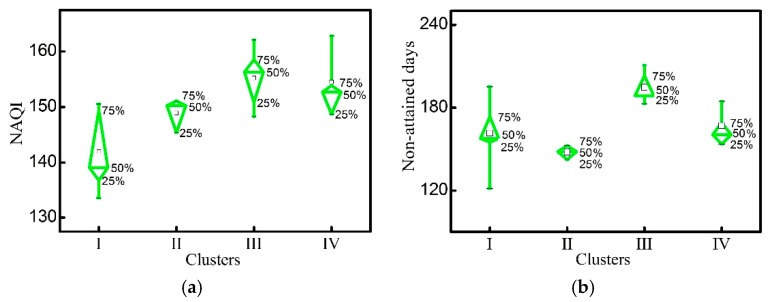
Diamond box plot of the NAQI (**a**) and non-attainment days (**b**) based on the dendrogram. The bottom and top of the green box represent the 25th and 75th percentile, and the bottom and top black line represent the minimum and maximum value, respectively. The hollow square represents the mean value.

The hierarchical clustering analysis identified four clusters beyond an Euclidean distance of 10.0 (Figure 7). Cluster I, mainly located in the south-central part of Henan Province, consisted of Xuchang, Zhumadian, Pingdingshan, Nanyang, Shangqiu, and Xinyang. This cluster had the lowest NAQI among the four clusters, and an intermediate average number of non-attainment days (160). In contrast, cluster II (Kaifeng, Hebi and Zhoukou) had a moderate mean NAQI value, and the lowest average number of non-attainment days (148). Cluster III, in the north central Henan Province (Zhengzhou, Xinxiang, Puyang, Luohe, and Anyang) had the largest mean NAQI and highest number of non-attainment days. Finally, cluster IV, in northwestern Henan Province (Luoyang, Sanmenxia, Jiaozuo, and Jiyuan) had a relatively high mean NAQI and 167 non-attainment days (Figure 8).

### 3.3. Relationships between Air Quality Index on Non-Attainment Days (NAQI) and Influencing Factors

We found significant relationships between the daily NAQI and average temperature, sunshine hours, average and maximum surface temperature that were generally negative in all cities throughout the study period, but positive relationships between the daily NAQI and average relative humidity, and average atmospheric pressure (Table 1). More sunshine and higher temperature are linked with vertical and horizontal turbulence, which results in greater dispersion and dilution of air pollutants [8]. Air is usually still when high-pressure systems are in place, which allows pollutants to accumulate. However, under low-pressure systems, the weather is often wet and air is moving, causing the dispersion and deposition of pollutants [20]. In terms of relative humidity, some studies have reported that high humidity conditions can promote the formation of nitrate and secondary organic aerosols [41], and that relative humidity is positively related to air pollution [36]. However, others have argued that pollutant particles can absorb more water, agglomerate, and fall to the ground more easily at a higher relative humidity [16,20]. There may be a threshold point in the relationship between relative humidity and air pollution. With a gradual increase in daily relative humidity, air pollution appears to first increase and then decrease [22]. In addition, we demonstrated that the maximum instantaneous wind speed contributed to the dispersion of air pollution in most cities of clusters I–III, which was in accordance with the results of Xu et al. [8] and Sun et al. [9]. No significant correlation occurred for most cities of cluster IV, probably due to the low wind speed influenced by topographic constraints. In contrast, air pollution was negatively related to maximum instantaneous wind direction for several cities across the central and northern regions, suggesting that the contribution of regional transport under the north wind cannot be ignored. Such results are consistent with expectation, given the serious pollution in the northern region [40]. These findings have important implications for air quality forecasting and control. No significant correlation was found between daily precipitation and the NAQI for all cities (Table 1).

Table 2 shows the effects of selected social and economic factors on the NAQI at the city level in Henan Province. The NAQI in Henan Province from 2015 to 2016 was significantly correlated with the gross domestic product per capita (correlation coefficient (*R*) = 0.43; *p* < 0.05). In the industry structure category, the proportion of primary industry was a significant determinant of NAQI, but the effects of proportion of secondary industry and tertiary industry were not obvious (*p* = 0.061, 0.373). Economic development has a strong effect on air pollution [9,14]. A higher proportion of primary industries and a lower proportion of secondary industries are favorable for improving air quality [8]. Compared with the value-added by construction per capita, value-added by industry per capita had a significantly positive relationship with the NAQI (*R* = 0.42; *p* < 0.05), which could be attributed to the fact that the added value by industry was about 10 times higher than that added by construction during the two years.

Economic growth is associated with an increase in energy consumption and the number of vehicles [8,25,30]. Total energy consumption per unit area was significantly related to NAQI (*p* < 0.01), especially coal, heat, and electricity consumption (*p* = 0.054, 0.045, 0.002). In Henan Province, coal consumption is enormous, accounting for 80.2% of the total energy consumption, causing serious coal smoke pollution [34]. Meanwhile, civil vehicles possession per unit area played an important role in air quality inequities. Cities with higher sedan and heavy truck per unit area values experienced more serious air pollution (*p* = 0.053, 0.014). Vehicle emissions have become one of the major sources of air pollution in Henan Province, with annual total emissions reaching 3.927 million tons in 2016, accounting for about 10% of the country’s total emissions [42]. Among vehicles, the light-duty gasoline-powered vehicles and heavy-duty diesel-powered vehicles may be the largest contributors [43].

Complex relationships among socioeconomic factors and NAQI were demonstrated among cluster types. NAQI was significantly correlated with civil vehicles possession (*p* < 0.05), total energy consumption (*p* < 0.05), and floor space under construction per unit area (*p* < 0.01) for cities of cluster I, but such a relationship was obvious for only floor space under construction (*p* < 0.01) and diesel fuel per unit area (*p* < 0.05) for cluster II. For cluster III, there was a significant relationship between the NAQI and coke per unit area. Additionally, cities in cluster IV, with more trucks, large passenger vehicles, and floor space under construction and floor space under completion per unit area experienced more severe air pollution. However, there were some degrees of uncertainty. For instance, in clusters III and IV, the mean proportions of secondary industry and energy usage per unit of gross domestic product were obviously higher than that for other cluster types. However, the relationships between the NAQI and proportion of secondary industry, and total energy consumption per unit area were not evident, probably due to the limitations of sample size and the diversity of emission characteristics in each cluster. These limitations are expected to be overcome in the future. 

## 4. Conclusions

We investigated the spatial and temporal variations of air pollution and its influencing factors for 18 cities in Henan Province from April 2015 to February 2017, with the goal of enhancing our understanding of the spatiotemporal characteristics and underlying mechanisms. Our results showed that ambient air pollution has become more serious in some cities, although various policies and actions have recently been implemented to control air pollution in Henan. Cities such as Hebi, Puyang, and Anyang experienced marked increases in both the NAQI and non-attainment days. The spatial autocorrelation analysis placed Anyang and Hebi in a high–high spatial cluster in 2016, indicating that air pollution in these cities could continue to be reduced by implementing joint measures. In addition, although PM_2.5_ remained the most frequent major pollutant in all cities, the major air pollutant structure changed greatly during the study period, posing a new challenge to urban air quality improvement. The meteorological factors including mean temperature, sunshine hours, average and maximum surface temperature were negatively related to the NAQI, while relative humidity and mean atmospheric pressure had positive impacts on the NAQI. Air pollution was greatly affected by socioeconomic factors. Generally, primary industry has a significant negative correlation with the NAQI, while secondary industry showed a positive correlation with the NAQI. Additionally, the energy structure, civilian vehicle composition, and construction factors had certain impacts on air pollution, with the magnitudes of the effects depending on the city. These findings enhance our understanding of the variation characteristics of regional air pollution, which is critical for guiding region-oriented policy making to reduce air pollution.

## Figures and Tables

**Table 1:** Meteorological factors associated with the air quality index on non-attainment days (NAQI) at the city level during 2015–2016. MWS, MWD, SH, AAP, AT, ARH, P, AST and MST denotes the daily maximum instantaneous wind speed and direction, sunshine hours, average atmospheric pressure, average temperature, and average relative humidity, precipitation, average and maximum surface temperature, respectively.

Cluster	City	Correlation Coefficient								
		MWS	MWD	SH	AAP	AT	ARH	P	AST	MST
I	Xuchang	−0.17 **	−0.19 **	−0.38 **	0.33 **	−0.43 **	0.26 **	−0.06	−0.44 **	−0.45 **
	Zhumadian	−0.17 **	0.06	−0.35 **	0.28 **	−0.37 **	0.30 **	0.02	−0.39 **	−0.42 **
	Pingdingshan	−0.18 **	−0.05	−0.36 **	0.25 **	−0.34 **	0.20 **	−0.06	−0.36 **	−0.35 **
	Nanyang	−0.15 **	−0.05	−0.30 **	0.33 **	−0.42 **	0.17 **	−0.08	−0.43 **	−0.41 **
	Shangqiu	−0.16 **	0.01	−0.28 **	0.28 **	−0.40 **	0.23 **	−0.04	−0.41 **	−0.41 **
	Xinyang	−0.17 **	0.07	−0.39 **	0.19 **	−0.32 **	0.33 **	0.04	−0.33 **	−0.39 **
II	Kaifeng	−0.21 **	−0.22 **	−0.43 **	0.30 **	−0.42 **	0.36 **	−0.04	−0.44 **	−0.47 **
	Hebi	−0.39 **	−0.07	−0.45 **	0.30 **	−0.42 **	0.36 **	−0.06	−0.44 **	−0.48 **
	Zhoukou	−0.04	−0.03	−0.29 **	0.26 **	−0.33 **	0.20 **	0.04	−0.35 **	−0.37 **
III	Zhengzhou	−0.28 **	−0.12 **	−0.37 **	0.33 **	−0.47 **	0.35 **	−0.06	−0.47 **	−0.46 **
	Xinxiang	−0.29 **	−0.04	−0.45 **	0.35 **	−0.48 **	0.34 **	−0.01	−0.50 **	−0.52 **
	Puyang	−0.37 **	−0.10	−0.37 **	0.34 **	−0.43 **	0.28 **	−0.05	−0.44 **	−0.46 **
	Luohe	−0.09 *	−0.09 *	−0.34 **	0.31 **	−0.42 **	0.17 **	−0.08	−0.42 **	−0.41 **
	Anyang	−0.34 **	−0.10 *	−0.43 **	0.38 **	−0.50 **	0.32 **	−0.04	−0.51 **	−0.54 **
IV	Luoyang	−0.06	−0.16 *	−0.37 **	0.34 **	−0.46 **	0.17 **	−0.07	−0.45 **	−0.44 **
	Sanmenxia	−0.04	0.07	−0.36 **	0.39 **	−0.48 **	0.08	−0.07	−0.49 **	−0.49 **
	Jiaozuo	−0.27 **	−0.06	−0.44 **	0.34 **	−0.48 **	0.38 **	−0.08	−0.48 **	−0.50 **
	Jiyuan	−0.04	−0.22 **	−0.33 **	0.24 **	−0.37 **	0.19 **	−0.08	−0.36 **	−0.36 **

** *p* < 0.01, * *p* < 0.05.

**Table 2:** Socioeconomic factors associated with the NAQI at the city level during 2015–2016. PCV, PPV, PLPV, PS, PT, PHT, POT means per unit area civil vehicle possession, passenger vehicle, large passenger vehicle, sedan, truck, heavy truck, and ordinary truck; PTEC, PCL, PCK, PDF, PH, PE denotes per unit area total energy consumption, coal, coke, diesel fuel, heat and electricity of industrial enterprises above a designated size; PFSCI and PFSCII shows per unit area floor space under construction and floor space under completion; PPI, PSI, PTI, PGDP, PVAI, and PVAC means the proportion of primary industry, secondary industry, tertiary industry, per capita gross domestic product, value-added by industry, and value-added by construction.

Factor	Variable	Correlation Coefficient				
		I	II	III	IV	Total
Economic structure	PGDP	0.66	0.97	0.01	−0.43	0.43 *
	PPI	−0.70	−0.86	−0.06	−0.16	−0.60 **
	PSI	0.70	0.49	−0.78	−0.19	0.38
	PTI	−0.31	−0.11	0.58	0.26	0.08
	PVAI	0.70	0.79	−0.31	−0.29	0.42 *
	PVAC	0.02	0.71	0.33	−0.57	0.35
Possession of civil vehicle	PCV	0.79 *	0.91	−0.03	0.85	0.40
	PPV	0.80 *	0.95	−0.01	0.83	0.39
	PLPV	0.60	0.94	−0.02	0.96 *	0.34
	PS	0.78 *	0.93	−0.01	0.78	0.39
	PT	0.67	-0.94	−0.35	0.95 *	0.43 *
	PHT	0.61	-0.88	−0.39	0.89	0.51 *
	POT	0.68	-0.92	−0.39	0.89	0.30
Energy consumption of industrial enterprises above a designated size	PTEC	0.87 *	0.93	0.72	0.11	0.60 **
	PCL	0.67	0.74	0.39	0.24	0.39
	PCK	0.30	0.32	0.88 *	−0.40	0.32
	PDF	0.79 *	1 *	−0.43	−0.81	0.13
	PH	0.64	0.61	−0.59	0.76	0.41 *
	PE	0.78 *	0.96	0.39	0.67	0.64 **
Floor space of buildings	PFSCI	0.89 **	1 **	0.02	0.94 *	0.38
	PFSCII	0.23	−0.03	−0.16	1 **	0.25

** *p* < 0.01, * *p* < 0.05.
